# Bulgarian General Practitioners’ Communication Styles about Child Vaccinations, Mainly Focused on Parental Decision Making in the Context of a Mandatory Immunization Schedule

**DOI:** 10.3390/healthcare11182566

**Published:** 2023-09-17

**Authors:** Veronika Dimitrova, Savina Stoitsova, Gergana Nenova, Maria Martinova, Milena Yakimova, Vanya Rangelova, Irina Georgieva, Ivo Georgiev, Stefka Krumova, Antoaneta Minkova, Nadezhda Vladimirova, Lubomira Nikolaeva-Glomb

**Affiliations:** 1 Department of Sociology, Sofia University, 1000 Sofia, Bulgaria; g.nenova@phls.uni-sofia.bg (G.N.); yakimova@phls.uni-sofia.bg (M.Y.); 2 Department of Epidemiology, National Centre of Infectious and Parasitic Diseases, 1504 Sofia, Bulgaria; stoitsova@ncipd.org (S.S.); ivo.georgiev@ncipd.org (I.G.); tonyminkova@ncipd.org (A.M.); nvladimirova@ncipd.org (N.V.); 3 Communities and Identities Department, Institute of Sociology and Philosophy at the Bulgarian Academy of Sciences, 1000 Sofia, Bulgaria; maria.martinova@ips.bas.bg; 4 Department of Epidemiology and Disaster Medicine, Medical University of Plovdiv, 4002 Plovdiv, Bulgaria; vanya.rangelova@mu-plovdiv.bg; 5 Department of Virology, National Centre of Infectious and Parasitic Diseases, 1504 Sofia, Bulgaria; irina.georgieva@ncipd.org (I.G.); skrumova@ncipd.org (S.K.); lubomira.nikolaeva.glomb@ncipd.org (L.N.-G.)

**Keywords:** vaccine hesitancy, general practitioners, communication styles

## Abstract

The communication practices of general practitioners in relation with vaccines have not been a topic of wide scientific interest. In this article, we outline them in the context of Bulgaria. A representative, cross-sectional, quantitative, face-to-face survey was conducted among 358 Bulgarian general practitioners in 2022 using simple random sampling. We conducted an exploratory factor analysis using questions about the role of the GPs, which measure models of communication. Based on the factor analysis, we distinguished four communication styles. They were called: active communicator, restrictive communicator, informing communicator, and strained communicator. One-way ANOVA and the T-test were carried out to explore the connections between factor scores (communication styles) and other variables. One of the most important results in the study was that the informing physician (emphasizing the choice of the parents) was the most common model in Bulgaria. This is somewhat contradictory, because of the mandatory status of most vaccines. We found connections between the communication styles and other variables—such as the type of settlement, having a hesitant parent in the practice, recommendations of non-mandatory vaccines, and experience with vaccine-preventable diseases. On the basis of the factor analysis and analysis of relationships with other variables, we reached the conclusion that in Bulgaria, hesitant parents are not sufficiently involved in active, effective communication about vaccines by GPs.

## 1. Introduction

Vaccination has made a significant contribution to global health, leading to a substantial reduction in the incidence of multiple diseases. At the same time, hesitancy towards vaccines within populations is a known driver of suboptimal vaccination coverage. Vaccine hesitancy (VH) has attracted increasing attention of the scientific community. In 2019, VH was recognized by the World Health Organization (WHO) as one of the key threats to global health [1]. VH is a widely studied phenomenon, and is inherent to the population [2,3,4]. Compared to other parts of the world, countries in the European Union (EU) are less vaccine-confident, and there is a trend towards decreasing confidence over time [5,6]. Eastern European countries are even more hesitant than the rest of the EU. Bulgaria is one of the countries in the EU with the lowest levels of trust in the safety and effectiveness of vaccines [5].

VH has also been identified in studies among healthcare workers [5,7,8,9,10,11,12]. For most general practitioners (GPs), regardless of their personal position on vaccines, it is an obligation to provide vaccine information to their patients. While there are studies on hesitancy among physicians as predictors of low vaccine recommendations, their communication practices have not been a topic of wide scientific interest [13], except in the context of evidence-based interventions such as the presumptive manner of recommendation [14,15,16,17] and the motivational interviewing approach [18,19,20]. At the same time, a number of studies have demonstrated that physicians, and in particular general practitioners, play a key role in the communication regarding vaccines, because they are a preferred information source for parents and can influence parental decision-making regarding vaccines [9,11,21,22,23,24,25,26,27].

This article presents results from a study which aimed to delve into this unexplored topic by focusing on the attitudes and communication styles of Bulgarian GPs. Our study consisted of two parts, the first of which focusing on attitudes towards vaccination and the second on communication styles. The first part, which focused on GP attitudes towards vaccination, was analyzed in another publication (in print). To summarize of the first part, vaccine confidence among GPs in Bulgaria is comparable to that of GPs in other European countries and higher than that of the general population, with 98% (95% Confidence Interval (CI) CI, 96–99%) of respondents strongly agreeing or agreeing that vaccines are important, 98% (95% CI, 96–99%) that vaccines are effective, and 95%, 95% CI, 93–97% that vaccines are safe. The results from the second part of the study, focusing on communication styles, are summarized in the current publication. The aims were threefold: to investigate communication practices, to propose a typology of communication styles, and to explore the relationships between these styles and other variables.

## 2. Materials and Methods

### 2.1. Overview of the Study Approach

This study utilized exploratory factor analysis, an analysis type widely accepted in sociological methodology to study social phenomena which are understudied and which are only beginning to be contextualized and characterized, such as GP communication styles. The approach employs (1) a pre-study preparation carried out in order to devise a theoretical framework of hypothetical communication styles, as well as questions designed to test this framework; (2) factorial analysis of the results performed to test whether the hypothetical communication styles explain the variance of styles detected in the study. The pre-study preparation is described in more detail below, and involves a review of key theoretical frameworks in the literature, corroborated with contextual knowledge from experts and previous field work conducted in Bulgaria in the same population. The analytical part is also described in more detail later in the text.

### 2.2. Population Surveyed and Procedure

A representative, cross-sectional, quantitative, face-to-face survey was conducted among Bulgarian general practitioners (GPs) in June–July 2022. The inclusion criterion for participation in the study was that the general practitioner had children enrolled in his practice (0–18 years old). This study excluded those who did not have children in their practices (0–18 years old). A simple random sample of 2002 GPs was randomly drawn from the comprehensive database of Bulgarian GPs available on the website of the Bulgarian National Health Insurance Fund (BNHIF), which included 3862 GPs. All 2002 GPs in the sample were contacted to verify contact details, check inclusion criteria, and confirm willingness to participate. A total of 993 GPs with children in their practices were successfully contacted, of whom 875 agreed to participate (88% acceptance rate). Among them, 358 GPs were randomly selected (simple random sample) for face-to-face interviews. This study aimed to interview 350 general practitioners for a representative sample from the entire database (3862 practitioners), with a confidence interval of 95%, a 5% margin of error, and a proportion of 50% of the population. Eight respondents were added to ensure coverage of 350 participants if data were missing and respondents needed to be deleted. For the studied 358 participants, the confidence interval was 95% and the error level was 4.9%. The survey design and questionnaire were developed by the study team, and data collection was conducted by an external contractor (Global Metrix, Sofia, Bulgaria). The interviewers received training on the questionnaire before conducting the face-to-face interviews.

All participants gave informed consent to participate voluntarily and confidentially in the study.

### 2.3. Questionnaire

The questionnaire was developed after a review of the relevant literature (summarized in more detail below), a preliminary study, and discussions with a multidisciplinary group of experts in public health and social science (authors of the publication). It was pilot-tested with 20 GPs before finalization and implementation. No variables were modified, and the variables are reported in this article. The questionnaire consisted of two parts, focusing on attitudes towards vaccination and communication styles. The first part, which focused on GP attitudes towards vaccination, has been analyzed elsewhere (publication in print). The second part, which is the focus of this publication, aimed to register how GPs communicate with parents regarding vaccines. The questions were designed to capture communication styles based on two theoretical models described in literature—the physician-centered and the patient-centered model [28]. In the physician-centered model, the practitioner leads the communication in a more directed and controlled way based on professional expertise. In the patient-centered model, the communication is focused on the needs and perspectives of the patients, and the emphasis is on the patient’s decision-making and responsibility [28].

Aspects of communication in the existing studies are addressed through: 1. the frequency of vaccine recommendations regarding non-mandatory vaccines; 2. acceptance of the role of vaccination by the GPs; and 3. the extent to which they feel comfortable providing information to their patients [9,29,30,31]. This is why, when the study team composed the questionnaire, only two questions from other studies were used (described below).

Based on these two key theoretical models; an additional review of qualitative studies (referenced further in this paragraph); and findings from a thematic analysis of a preliminary study with 15 semi-structured interviews with GPs, which was focused on attitudes towards vaccination and was carried out previously by members of the study team, we compiled four models of communication styles to be tested via the questionnaire. The models were explored through a set of statements, and responses to these statements were rated on a 5-point Likert scale. The first model to be tested was called the active communicator, a patient-centered model in which the GP plays the role of active communicator. Three statements were included—“My role is to explain to parents the benefits of vaccines, even if they initially hesitate or refuse to vaccinate their children” (the question used in [30]); “I explain to parents the side effects of vaccines, as well as the risks of vaccine-preventable diseases”; and “I try to understand parents’ hesitations”. The second model to be tested was called the restrictive communicator, a physician-centered model in which GPs refuse to enroll hesitant parents in the practice and withdraw from communication with them [32,33]. Three statements were included—“I do not accept in my practice parents who do not wish to vaccinate their children”; “Parents who hesitate and delay vaccines hinder my work”; and “In my practice as a general practitioner, I do not allow children to remain unvaccinated due to a parent’s refusal”. The third model to be tested is that of the informing communicator—a style that is an excessive form of the patient-centered model, in which the individual choice of the parent to vaccinate their child takes on a central role [8,11,13]. Three statements were included—“The decision to vaccinate is the parents’ responsibility”; “I am pushing for vaccinations” (reverse meaning will be explored); and “I inform the parents, but the choice is theirs”. The fourth model to be tested was called the strained communicator, which aims to explore the tension felt by the GPs. Three statements were considered in three domains: (1) the extent to which GPs feel comfortable communicating: “I generally feel comfortable in front of parents when explaining about vaccines” (reverse meaning) (questions about comfortability with giving explanations were used in [9,31,34]); (2) the tension felt by GPs: “GPs do not have a choice to administer or not to administer vaccines—this sometimes makes communication with parents tense” (as documented in [26]); and (3) vaccination as an administrative burden: “I don’t have enough time to spend explaining to parents”.

Other questions included in the questionnaire concerned practice characteristics, such as size, number of children in the practice, type (individual or group), settlement (capital, regional city, small town or village), age and gender of the GP, and specialty. It also included parental hesitancy and attitudes towards vaccines as perceived by the GP, such as how often parents of children from the GP practice have doubts regarding the administration of vaccines; whether there has been a change in parents’ vaccine attitudes about mandatory childhood vaccines since the start of the COVID-19 pandemic; and experience with diseases and side effects. The latter includes the questions of whether there have been cases of illnesses due to diseases against which mandatory immunizations from the children’s immunization calendar in Bulgaria are currently applied; if there are cases of serious adverse effects following compulsory vaccination of children that were potentially associated with hospitalization or disability; and communication and recommendations of non-mandatory vaccines, such as whether the respondent perceives overall difficulties communicating with parents regarding the vaccination of their children and how often the respondent recommends non-mandatory vaccinations.

### 2.4. Methods of Analysis

Even though simple random sampling is the strongest methodology and provides the opportunity to generalize conclusions to the overall studied population, we performed an additional final check for representativeness, comparing the distribution by type of settlement of the sample of respondents to the same distribution of the general GP population (as obtained from the database of the BNHIF).

Factor analysis was chosen as a method because we wanted to explore the underlying dimensions (styles of communication) between the 12 items of the questionnaire measuring the communicational practices of GPs. “I do not wish to answer” was recoded as a missing variable for all 12 items regarding communication. We conducted an exploratory factor analysis using the questions about the role of GPs in communication. Exploratory factor analysis is a widely accepted method in the early stages of instrument development to measure latent factors. To perform the initial factor analysis, we used the principal factor method. All items with loadings higher than 0.45 were accepted. As indicated above, for some statements, the reverse meaning was explored in the analysis. This means that in a statement including, for example, “I don’t have time”, exploring the reverse meaning refers to statistically loading the statement in the model in the other direction (i.e., those who strongly disagree with the statement “I don’t have time” are assumed in the analysis to be respondents who strongly agree with the statement “I have time”).

After the factor analysis was performed, mean factor scores for each GP and each factor were calculated as averages of the corresponding variables. This allows for (1) assessment of the extent to which each GP accepts a given model of communication (factors) and (2) testing of relationships between the factors as outcomes (dependent) and the other independent variables.

One-way ANOVA and the T-test were chosen to test the relationships between quantitative, dependent variables (factor scores for each variable) and qualitative, independent ones. One-way ANOVA and the T-test were carried out by taking the assigned factor mean scores as dependent variables, and taking 13 independent variables capturing the characteristics of the GP practice, parental attitudes towards vaccines as perceived by the GP, experience with vaccine-preventable diseases and side effects, difficulties in communication, and the GP’s propensity to recommend non-mandatory vaccines.

## 3. Results

The test to assess the representativeness of the sample showed that the distribution of the sample by type of settlement was almost identical to the distribution of the general GP population by type of settlement. More specifically, 18% of the surveyed GP population worked in the capital (Sofia), 41% in regional capital cities, 27% in small towns, and 13% in villages. The corresponding distributions in the general population of GPs were 19%, 42%, 27%, and 13%. The post hoc test for representativeness, as expected, demonstrated that the simple random sample drawn in this study can be considered representative, and the results can be viewed as generalizable to the overall studied population.

The characteristics of the GPs are presented in Table 1.

### 3.1. Factor Analysis

When the principal factor method for factor analysis was used, the scree plot break occurred at the fourth factor, which also corresponds to the theoretical number of factors. Therefore, we forced the creation of four factors, explaining 63.62% of the total variance (Table 2). The models of communication (or Factors) could coexist and overlap.

Our analysis revealed four factors. The Kaiser–Meyer–Olkin Measure of Sampling Adequacy was 0.700, and the result was acceptable.

As can be seen from the matrix, the factors almost corresponded to the theoretical model described in the Questionnaire section (Table 3).

Factor 1 is patient-centered approach with emphasis on communication and insistence on vaccines (active communicator). Factor 1 includes statements supporting the role of an active communicator. The following items are considered for Factor 1: “My role is to explain to parents the benefits of vaccines, even if they initially hesitate or refuse to vaccinate their children” (0.741); “I explain to parents the side effects of vaccines, as well as the risks of vaccine-preventable diseases” (0.679); “I am pushing for vaccinations” (0.552); “I generally feel comfortable in front of parents when explaining about vaccines” (0.471); “I don’t have enough time to spend explaining to parents” (−0.598) (reverse meaning, negative loading) (Table 3). Factor 1 involves suggesting to the patient that the vaccination is in their best interest.

Our research showed that 48.6% of the physicians in the sample had a mean score lower than 1.5 (ranging from 1 to 5 corresponding to the Likert scale; this means that the respondents answered the statements included in the factor primarily with 1—“strongly agree”), and could be considered to strongly apply this model of communication.

Factor 2 is a physician-centered model in which refusal to vaccinate on the side of the parent leads to some form of dismissal from the GP (restrictive communicator). Factor 2 includes two statements: “I do not accept in my practice parents who do not wish to vaccinate their children” (0.85) and “In my practice as a general practitioner, I do not allow children to remain unvaccinated due to a parent’s refusal” (0.86).

According to our study, only 27.4% of the physicians had a mean score lower than 1.5, and these could be considered to strongly apply this model.

Factor 3 is the excessive form of the patient-centered approach: that of the informing physician (informing communicator). It consists of three statements: “I try to be understanding of parents’ hesitations” (0.51); “The decision to vaccinate is the parents’ responsibility” (0.786); and “I inform the parents, but the choice is theirs” (0.735).

According to the results, this model is widespread among the Bulgarian GPs, as 67.8% of the GPs achieved a mean value lower than 1.5.

Factor 4 is a strained model of communication (strained communicator). Factor 4 includes two statements: “GPs do not have a choice to administer or not to administer vaccines—this sometimes makes communication with parents tense” (0.768) and “Parents who hesitate and delay vaccines hinder my work” (0.701).

A total of 30.02% of the GPs had a mean value lower than 1.5 and could be considered to adopt this model.

### 3.2. Associations between Mean Factor Scores and the Profile of the Respondent

The mean factor scores were calculated, and the ANOVA analysis and T-test were performed. The four factors revealed in the factor analysis and their significance scores (Sig) showed complex relations with the profiles of the respondents (Table 4, Table 5, Table 6 and Table 7). Note that a significance score of <0.05 is considered to show a significant association.

#### 3.2.1. Relationships with General Characteristics of the Practice

In the analysis, we found connections between the communication styles and the type of settlement (Table 4). It should be noted that these patterns probably highlight the specifics of communication in different types of settlements. With Factor 1 (active communicator), the relationship was not significant (sig = 0.09), although from the means, it appears that practitioners in small towns (M = 1.4737, SD = 0.30604, Supplementary File) were the most likely to adopt this model of active communication and vaccination push. With Factor 2 (restrictive communicator) is the results were different—the relationship was significant (sig < 0.001), and we found that practitioners in regional cities (M = 2.2899, SD = 1.20342) and the capital (M = 2.6587, SD = 1.27894) were the most likely to adopt the restrictive communicator model. This probably reflects competition between GPs in different localities. Factor 3 (informing communicator) was also linked to the type of settlement (sig < 0.001). It was most often adopted in small towns (M = 1.1803, SD = 0.43122) and villages (M = 1.1884, SD = 0.635), in contrast to the capital (M = 1.7374, SD = 1.7374) and regional cities (M + 1.6643, SD = 0.90105). The association between Factor 4 and the type of settlement was also significant (sig < 0.001). The mean scores of Factor 4 (strained communicator) demonstrated that this type of communication was used by GPs more often in the capital (M = 2.1094, SD = 0.85202), in regional towns (M = 2.3406, SD = 1.06192), and in villages (M = 2.2391, SD = 1.16304) compared with small towns (M = 2.9062, SD = 1.17274). These scores probably reflect the problems with the implementation of an immunization plan in each of these types of settlements, which may possibly depend on differences in the characteristics of the population being serviced.

The relationship seems to reflect the specifics of practices in different types of settlement, and it is possible that other variables which were not studied are also related to characteristics of the communication in various localities, such as competition between practices, specifics of the population, and long-standing acquaintance between the doctor and the parents.

There was a significant relationship with the specialty (Table 5). We found significant associations between a pediatric specialty and Factor 2 (sig < 0.05) and Factor 3 (sig < 0.01). It seems that pediatricians more often adopt a restrictive communicator model (Factor 2) (M = 2.6075, SD = 1.37365) and less often adopt an informing communicator model (Factor 3) (M = 1.6972, SD = 0.9434). From here, we can also pose a hypothesis for next study—highly popular practices more often use a communication model related to dismissal of parents and accept patients’ decisions less often. The associations between a specialty in general medicine and Factor 2 and Factor 3 were significant (sig < 0.05, sig < 0.01) (Table 5). From the mean factor scores and the standard deviation, we can state (Supplementary File) that physicians with specialties in general medicine adopt the opposite model—those with this specialty more often use an informing communicator model (Factor 3) (M = 1.59, SD = 0.79994), and less often exhibit a restrictive communicator model (Factor 2) (M = 2.7733, SD = 1.32545).

We did not find significant relationships among the type of practice, gender (except for Factor 2—restrictive communicator (sig < 0.05), where women more often adopt this model of communication) (M = 2.686, SD = 1.29014), age, size of the practice, or number of children in the practice (Table 4 and Table 5).

#### 3.2.2. Relationships with Perceived Parental Hesitancy and Vaccine Recommendations

There were associations found between the perception of physicians that there were hesitant parents in their practice and Factor 1 (sig = 0.001), Factor 2 (sig < 0.05), and Factor 3 (sig < 0.001) (Table 5). Physicians adopting an active communicator model (Factor 1) were more likely to say that there were no parents in their practice who were hesitant (M = 1.4707, SD = 0.48652) (Supplementary File).

Physicians adopting a restrictive communicator model (Factor 2) more often stated that the parents in their practice had hardly any doubts about vaccines (“Yes, rarely”—M = 2.7201, SD = 1.25523; “No”—M = 2.8043, SD = 1.41374). This can be explained by the practice of dismissal of hesitant parents (Table 6).

We found a relationship between experience with vaccine-preventable diseases and Factor 3 (informing communicator) (sig < 0.01) (Table 6); physicians adopting this model were more likely to have had no experience with vaccine-preventable diseases (M = 1.4417, SD = 0.765)

There was a relationship between Factor 4 (strained communicator) and perception of change in the attitudes about mandatory childhood vaccines since the start of the COVID-19 pandemic (sig < 0.001) (Table 6). Physicians who perceive vaccination as a burden and source of tension have more experience with communication with patients whom they perceive as hesitant. GPs who fell into this pattern were more likely to report that there has been a change in vaccine attitudes among parents regarding mandatory childhood vaccines since the start of the COVID-19 pandemic—they were more likely to report that there is more parental hesitancy (“Yes, there are more hesitant parents”—M = 2.0189, SD = 0.88889) (Table 6).

In the analysis, this study revealed associations between communication practices and communication styles. Having difficulties communicating with parents is associated with Factor 1 (sig = 0.001) and Factor 4 (sig < 0.001). Those adopting the active communicator model (Factor 1) more often stated that they have no problems with communicating with parents about vaccines (Answer “I do not experience any difficulties in communicating with parents”, M = 1.4831, SD = 0.51506). Those who fell into the strained communicator (Factor 4) model were also more likely to say that they have difficulty communicating with parents (“To a great extent”—M = 1.4706, SD = 0.92653; “To some extent”—M = 2.1207. SD = 0.82881; “To a small extent”—M = 2.1881, SD = 0.89698).

The recommendation of vaccines not included in the mandatory immunization schedule was linked to Factor 1 (sig < 0.001), Factor 2 (sig < 0.05), and Factor 4 (sig < 0.05). Those adopting the active communicator model more often recommended non-mandatory vaccines (“Always”—M = 1.4588, SD = 0.35554; “Often”—M = 1.5567, SD = 0.49475). Physicians using the restrictive communicator model (Factor 2) recommended vaccines always, often, and rarely (“Always”—M = 2.6893, SD = 1.38641; “Often”—M = 2.9444, SD = 1.26251; “Rarely”—M = 2.5781, SD = 1.33992). Those who adopted the strained communicator model (Factor 4) were the least likely to say that they always or often recommend non-mandatory vaccines (“Always”—M = 2.6667, SD = 1.09303; “Often”—M = 2.4387, SD = 1.09505).

## 4. Discussion

The reported results are part of a representative survey carried out in 2022 in Bulgaria, which aimed to investigate (1) vaccine confidence among general practitioners and (2) communication practices among GPs in the country. The first part of the survey confirmed the results from other studies that vaccine confidence among Bulgarian GPs is relatively high, and is comparable to the confidence measured among GPs in other European countries (data to be published) [5]. However, research shows that level of hesitancy among the Bulgarian population is one of the highest in Europe [5]. It seems that, along with demographic characteristics linked to the low vaccine confidence in the general population, such as age, gender, and education, other factors should be sought to explain this phenomenon. This is the reason why the communication practices of GPs in Bulgaria are the focus of the present study. In this article, we summarized the results from the communication practices component of the survey. We proposed theoretical models of the communication between patients and GPs, which we then tested through the survey and application of factor analysis. We also explored the relationships between models of communication (factors) and other variables in this study.

In this study, we have demonstrated that the predominant model of communication between GPs and parents regarding vaccines in Bulgaria is patient-centered and focused on parental decision-making, even though communication takes place in the context of a mandatory immunization schedule, and a physician-centered approach may have been expected instead. This finding may be a manifestation of the conflict arising in dealing with a hesitant population, and of the roles that GPs assign to their patients in decision making. We can assume that in the physician-centered model, social control is in the hands of the professionals, while in the patient-centered model, social control is in the hands of the patients [35]. But we may also suppose that, due to the institutional positions of GPs in the primary health care sector, social control can also dominantly belong to patients. Although not much quantitative research has been conducted on communication styles, there are at least a few significant qualitative studies which outline the modification of the general models in relation to vaccines [7,24,26]. Some authors consider that the patient-centered model is a manifestation of the modern tendency toward personalized healthcare [26]. The implementation of mandatory vaccination schemes is in direct conflict with this model, i.e., the premise for decision-making and the possible tensions arising from it. This tension is reflected at the level of the relationship between the GP and parents of the patients in cases of childhood immunizations, but also at the level of the role that the general practitioner himself assumes.

Factor 1 (active communicator) suggests that vaccination is in the patient’s best interest and is not an administrative burden for GPs. These are the reasons for GPs to undertake active communication. We can suggest that those accepting this model are strong advocates of vaccines. The relationships between Factor 1 and other items from the questionnaire demonstrate that physicians adopting this model are more likely to have no experience with hesitant parents, report no communicational problems with the parents, and recommend additional vaccines more often. This can mean that GPs using this model are addressing vaccine hesitancy among their patients well, or that they are not sensitive to vaccine hesitancy among parents.

Factor 2 (restrictive communicator) suggests that physicians are strong supporters of vaccines, but their model emphasizes dismissal, not communication. This practice of dismissal has been documented not only for Bulgaria, but for other countries as well [32,33]. There are consistent links between Factor 2 and the type of practice—practitioners from regional cities with pediatric specialties adopt this model more often, and those specializing in general medicine adopt it less often. These characteristics lead us to the possible hypothesis that more desirable practices use this model of communication more often. It possibly explains why those GPs report more often that they rarely have vaccine-hesitant parents.

Factor 3 (informing communicator), which is also the dominant model in Bulgaria, demonstrates the behavior of general practitioners regarding the mandatory immunization calendar with the imposition of the idea of parental decision making. Parental expectations probably play a key role in the adoption of this model. Relationships between the type of settlement and Factor 3 show that this model is more strongly adapted in small towns and villages. Relationships with specialties demonstrate that pediatricians accept this model less often, and GPs with specialties in general medicine assume this model more often. GPs who follow this model are more likely to have no experience with vaccine-preventable diseases. It is likely that this approach is used more by GPs who share some of the hesitancy with their patients, as well as by GPs whose practices are more at risk of patient withdrawal. Here, vaccine intake is treated as an individual choice of the parent, as has been demonstrated in other studies [8,11,13].

Factor 4 (strained communicator) shows that in conditions of increasing hesitancy, GPs feel tension. Physicians who practice in the capital and in villages adopt this model more often. This probably indicates localities where there are more vaccine-hesitant parents, as well as difficulties with the administration and implementation of the Bulgarian mandatory childhood immunization schedule. This model should be enriched with facts about the social control over the practice—if social control belongs to the parents, it means that postponing or refusing vaccines emotionally burdens GPs and can possibly endanger the practice. This is demonstrated in the research of Neufeind and colleagues—that mandatory approaches are an expected burden on doctor–patient relationships [36]. Opel and colleagues, in their innovative research based on conversational analysis, indicated that only half of doctors continue to insist on vaccinations after engaging in difficult conversations and emphasized that “Engaging in conflict with VHPs [vaccine hesitant parents] takes an emotional toll on providers” [16]. It should also be assessed to what extent the practice itself is under threat due to the possibility of patient withdrawal. There is no clue as to whether the tension is felt because of the presumed role of GPs in mandatory vaccination (insisting on vaccination) or because of the extended patient-centered approach (I am pushed to follow the schedule, but patients want otherwise). It is possible that respondents who use this approach are physicians with problems with the implementation of the Bulgarian immunization schedule for children.

The results show that active communication styles are rarely used because of their restrictive style, withdrawal of responsibility, or perceived tensions. Similar contradictions (regarding the role about vaccines, opinions, and communicational behavior) and perceived tensions have been demonstrated in other studies [8,23,37,38].

The following limitations should be mentioned. First, the proposed design of the study reported in this article is new, and this restricts the possibility of comparison with other countries, as the phenomenon is understudied not only in Bulgaria, but also elsewhere. Second, although in the design of the study we attempted to use questionnaires that have been used in other studies and to compose new ones mainly in the field of communication, the questionnaire was not validated. This is because we wanted to investigate a problem that has not been well addressed in the existing literature. Third, in the questionnaire, we investigated only vaccination in general, and did not include specific types of vaccines. It is known that attitudes and practices vary by vaccine. Fourth, the participants responded to the questionnaire based on their self-reported behaviors. Even though the questionnaire was anonymous, reporting bias or social desirability bias cannot be excluded. Fifth, the participation in the study was voluntary (88% response rate). It is also possible that people who were interested in and concerned about the present issue of VH might have been more inclined to respond to this questionnaire. Sixth, although a simple random sample was used and a post hoc test for representativeness was performed (based on the type of locality in which the GPs practiced), our sample may not have captured rarer characteristics of the study population that are specific to certain smaller sub-groups. Nonetheless, this is the first representative study in Bulgaria to investigate GPs’ communication styles, and despite its limitations, it can be used as a basis for evidence-based recommendations and as a starting point for future studies.

## 5. Conclusions

Our research attempts to address the gap of existing knowledge on communication styles regarding mandatory child immunizations. On one hand, some of the proposed models of communication need to be improved, enriched, and refined, but factor analysis shows that they are acceptable. On the other hand, we reached the conclusion that in Bulgaria, hesitant parents are not sufficiently involved in active communication about vaccines by GPs. This means that most of the models of communications are formed on the basis of avoiding demanding patient–provider relationships (except for Factor 1) [20], which can possibly lengthen the communication [38] and enhance the feelings of administrative burden and tension.

The communication styles of GPs are not well documented in the existing literature on hesitancy. Little is known about how general practitioners cope in situations of increasing hesitancy among parents. We believe that our approach could be used and refined in other studies on basic patterns of communication. Although the knowledge on this topic is scarce, our results are consistent with those of other studies. Although the proposed model appears to be applicable, it should be noted that perhaps some of the statements should be refined and further developed to better capture physicians’ communication styles and suggested roles regarding the mandatory vaccination of children.

## Figures and Tables

**Table 1:** Personal and professional characteristics of the participants of the study.

Variable	Results
Personal characteristics	
Age median (25th percentile; 75th percentile)	58 years old (52 years old; 63 years old)
Gender, n (%)	
Male	103 (28.8)
Female	255 (71.2)
Specialty *, n (%)	
General Medicine	230 (64.2)
Pediatrics	113 (31.6)
Internal Medicine	87 (24.3)
Other	12 (3.4)
Number of specialties, n (%)	
One	281 (78.5)
Two or more	77 (21.5)
Location of practice, n (%)	
Capital	66 (18.4)
Regional city	147 (41.1)
Small town	98 (27.4)
Village	47 (13.1)
Professional characteristics	
Type of practice, n (%)	
Solo practice	318 (88.8)
Group practice	40 (11.2)
Number of patients in the practice, n (%)	
1–1000	63 (17.6)
1001–2000	175 (48.9)
2001–3000	88 (24.6)
3001–4000	15 (4.2)
>4001	11 (3.1)
No information	6 (1.7)
Number of children in the practice (aged 0–18 years old), n (%)	
1–200	120 (33.5)
201–400	70 (19.6)
401–600	65 (18.2)
601–1000	68 (19.0)
>1001	30 (8.4)
No information	5 (1.4)

* Option to list more than one specialty.

**Table 2:** Total variance, explained.

Component	Initial Eigenvalues		
	Total	% of Variance	Cumulative %
Factor 1	2.855	23.791	23.791
Factor 2	2.229	18.576	42.366
Factor 3	1.561	13.009	55.375
Factor 4	0.989	8.245	63.62

**Table 3:** Rotated component matrix.

		Factor 1	Factor 2	Factor 3	Factor 4
1	I generally feel comfortable in front of parents when explaining about vaccines	0.471 *	0.295	0.143	−0.463
2	I don’t have enough time to spend explaining to parents	−0.598 *	0.327	0.254	0.353
3	GPs do not have a choice to administer or not to administer vaccines—this sometimes makes communication with parents tense	−0.027	−0.041	0.138	0.768 *
4	In my practice as a general practitioner, I do not allow children to remain unvaccinated due to a parent’s refusal	0.034	0.86 *	−0.09	−0.1
5	Parents who hesitate and delay vaccines hinder my work	0.271	0.378	−0.124	0.701 *
6	I do not accept in my practice parents who do not wish to vaccinate their children	−0.071	0.85 *	−0.091	0.135
7	My role is to explain to parents the benefits of vaccines, even if they initially hesitate or refuse to vaccinate their children	0.741 *	−0.015	0.302	0.066
8	I explain to parents the side effects of vaccines, as well as the risks of vaccine-preventable diseases	0.679 *	0.018	0.423	0.078
9	I try to understand parents’ hesitations	0.298	0.003	0.51 *	−0.343
10	I inform the parents, but the choice is theirs	0.253	−0.114	0.735 *	0.09
11	I am pushing for vaccinations	0.552 *	0.485	0.137	0.165
12	The decision to vaccinate is the parents’ responsibility	0.041	−0.031	0.786 *	0.046

* The item loads in the factor.

**Table 4:** Relationships between factor mean scores and general characteristics of the practice. ANOVA analysis—only levels of significance are displayed in the table. Mean factor scores and standard deviations are displayed in the Supplementary File.

	Factor 1 (Active Communicator)	Factor 2 (Restrictive Communicator)	Factor 3 (Informing Communicator)	Factor 4 (Strained Communicator)
Type of settlement, Sig	0.09	0.000	0.000	0.000
Age, Sig	0.967	0.478	0.379	0.107
Size of the practice, Sig	0.665	0.795	0.827	0.32
Percentage of children in practice, Sig	0.553	0.054	0.125	0.563

**Table 5:** Relationships between factors’ mean scores and general characteristics of the practice. T-test—only levels of significance are displayed in the table. Mean factor scores and standard deviation are displayed in the Supplementary File.

	Factor 1 (Active Communicator)	Factor 2 (Restrictive Communicator)	Factor 3 (Informing Communicator)	Factor 4 (Strained Communicator)
Type of practice	0.911	0.072	0.812	0.057
Gender, Sig	0.575	0.02	0.311	0.677
Specialty—pediatric, Sig	0.923	0.035	0.002 *	0.796
Specialty—internal medicine, Sig	0.707	0.635	0.148	0.238 *
Specialty—general medicine, Sig	0.482	0.004	0.00 *	0.244

* Equal variances not assumed.

**Table 6:** Parental hesitancy and attitudes towards vaccines. ANOVA analysis—only levels of significance are displayed in the table. Mean factor scores and standard deviation are displayed in the Supplementary File.

	Factor 1 (Active Communicator)	Factor 2 (Restrictive Communicator)	Factor 3 (Informing Communicator)	Factor 4 (Strained Communicator)
Do the parents of children from your practice have doubts regarding the administration of vaccines from the mandatory children’s immunization calendar? Sig	0.001	0.013	0.109	0.000
In your practice as a General Practitioner in the last 5 years, have there been any cases of illnesses from diseases against which mandatory immunizations from the children’s immunization calendar in Bulgaria are currently applied? Sig	0.269	0.487	0.003	0.227
In your practice as a GP in the last 5 years, have there been any cases of serious adverse effects following compulsory vaccination of children that were potentially associated with hospitalization or disability? Sig	0.907	0.126	0.296	0.15
Has there been a change in parents’ vaccine attitudes about mandatory childhood vaccines since the start of the Covid-19 pandemic? Sig	0.216	0.202	0.78	0.000

**Table 7:** Communication practices (difficulties in communication and recommendations of non-mandatory vaccines); ANOVA analysis.

	Factor 1 (Active Communicator)	Factor 2 (Restrictive Communicator)	Factor 3 (Informing Communicator)	Factor 4 (Strained Communicator)
To what extent do you have difficulty communicating with parents regarding the vaccination of their children? Sig	0.001	0.105	0.696	0.000
How often do you recommend vaccines that are NOT included in the mandatory immunization schedule? Sig	0.000	0.034	0.437	0.022

## Data Availability

The data presented in this study are not available. The data are not publicly available due to ethical reasons.

## Supplementary Materials

The following supporting information can be downloaded at: https://www.mdpi.com/article/10.3390/healthcare11182566/s1.
