# Supplementary material for: Bulgarian General Practitioners’ Communication Styles about Child Vaccinations, Mainly Focused on Parental Decision Making in the Context of a Mandatory Immunization Schedule
Source: Healthcare (Basel). 2023 Sep 17;11(18):2566. doi: 10.3390/healthcare11182566 (PMC10531209; doi:10.3390/healthcare11182566)
Supplement: Supplementary file 1 [file healthcare-11-02566-s001.zip › healthcare-2588864-supplementary.pdf]

Table S1. Questionnaire

**Profile of the Respondent**	
**What is your gender?**	Male<br>Female<br>Other
**What is your age?**	number
**What is the number of patients in your practice?**	number
**Haw many children you have enrolled in your practice?**	number
**Where your practice is mainly settled?**	Capital city<br>Regional city<br>Small town<br>Village
**What is your practice?**	Solo practice<br>Group practice
**What is your specialty?**	General Medicine<br>Pediatrics<br>Internal Medicine<br>Other. Please, specify…
**TRUST**	
**Please share how much you trust the following factors related to vaccines from the children's immunization calendar of the Republic of Bulgaria.**	
**I have trust in the information on vaccines from official authorities (WHO, ECDC)**	5 point Likert Scale, ranging from Strongly agree to Strongly disagree, option for "I do not wish to answer"
**I have trust in the information on vaccines from Bulgarian official authorities (MoH and others)**	5 point Likert Scale, ranging from Strongly agree to Strongly disagree, option for "I do not wish to answer"
**I have trust in the information on vaccines from pharmaceutical companies**	5 point Likert Scale, ranging from Strongly agree to Strongly disagree, option for "I do not wish to answer"
**I have trust in the information on vaccines from other GPs**	5 point Likert Scale, ranging from Strongly agree to Strongly disagree, option for "I do not wish to answer"
**I have trust in the practices on vaccines from official authorities (WHO, ECDC)**	5 point Likert Scale, ranging from Strongly agree to Strongly disagree, option for "I do not wish to answer"
**I have trust in the practices on vaccines from Bulgarian official authorities (MoH and others)**	5 point Likert Scale, ranging from Strongly agree to Strongly disagree, option for "I do not wish to answer"

**I have trust in the practices on vaccines from pharmaceutical companies**	5 point Likert Scale, ranging from Strongly agree to Strongly disagree, option for "I do not wish to answer"
ATTITUDES TOWARDS VACCINES	
**And now let's talk about the vaccines from the mandatory children's immunization calendar. Can you share your overall assessment of the following statements:**	
**Vaccines are important for children to have**	5 point Likert Scale, ranging from Strongly agree to Strongly disagree, option for "I do not wish to answer"
**Vaccines are safe**	5 point Likert Scale, ranging from Strongly agree to Strongly disagree, option for "I do not wish to answer"
**Vaccines are effective**	5 point Likert Scale, ranging from Strongly agree to Strongly disagree, option for "I do not wish to answer"
**It's good the vaccines are mandatory**	5 point Likert Scale, ranging from Strongly agree to Strongly disagree, option for "I do not wish to answer"
**All vaccines from the schedule are important**	5 point Likert Scale, ranging from Strongly agree to Strongly disagree, option for "I do not wish to answer"
**I generally approve the Bulgarian immunization calendar**	5 point Likert Scale, ranging from Strongly agree to Strongly disagree, option for "I do not wish to answer"
**Generally, the diseases for which there are mandatory vaccines are dangerous**	5 point Likert Scale, ranging from Strongly agree to Strongly disagree, option for "I do not wish to answer"
EXPERIENCE AND HESITANCY AMONG PARENTS	
**In your practice as a General Practitioner in the last 5 years, have there been any cases of illnesses from diseases against which mandatory immunizations from the children's immunization calendar in Bulgaria are currently applied?**	Yes, without administered vaccine<br>Yes, with administered vaccine<br>No<br>I con not answer
**In your practice as a GP in the last 5 years, have there been any cases of serious adverse effects following compulsory vaccination of children that were potentially associated with hospitalization or disability?**	Yes<br>No<br>I cannot answer
**Do the parents of children from your practice have doubts regarding the administration of vaccines from the mandatory children's immunization calendar?**	Yes, often<br>Yes, rarely<br>No<br>I cannot answer

**Has there been a change in parents' vaccine attitudes about mandatory childhood vaccines since the start of the Covid-19 pandemic?**	Yes, there are less vaccine hesitant parents<br>Yes, there are more vaccine hesitant parents<br>No<br>I cannot answer
**Are there children in your practice who have missed mandatory vaccinations for which they are eligible due to delay or refusal?**	Yes, due to refusal<br>Yes, due to delay<br>Yes, due to medical reasons<br>No<br>I cannot answer
**To what extent do you have difficulty communicating with parents regarding the vaccination of their children?**	To a great extent<br>To small extent<br>I do not experience any difficulties in communicating with parents<br>I connot answer
**COMMUNICATION**	
**How much do you agree with the following statements:**	
**I generally feel comfortable in front of parents when explaining about vaccines**	5 point Likert Scale, ranging from Strongly agree to Strongly disagree, option for "I do not wish to answer"
**I don't have enough time to spend explaining to parents**	5 point Likert Scale, ranging from Strongly agree to Strongly disagree, option for "I do not wish to answer"
**GPs do not have a choice to administer or not to administer vaccines – this sometimes makes communication with parents tense**	5 point Likert Scale, ranging from Strongly agree to Strongly disagree, option for "I do not wish to answer"
**In my practice as a general practitioner, I do not allow children to remain unvaccinated due to a parent's refusal**	5 point Likert Scale, ranging from Strongly agree to Strongly disagree, option for "I do not wish to answer"
**Parents who hesitate and delay vaccines hinder my work**	5 point Likert Scale, ranging from Strongly agree to Strongly disagree, option for "I do not wish to answer"
**I do not accept in my practice parents who do not wish to vaccinate their children**	5 point Likert Scale, ranging from Strongly agree to Strongly disagree, option for "I do not wish to answer"
**My role is to explain to parents the benefits of vaccines, even if they initially hesitate or refuse to vaccinate their children**	5 point Likert Scale, ranging from Strongly agree to Strongly disagree, option for "I do not wish to answer"
**I explain to parents the side effects of vaccines, as well as the risks of vaccine-preventable diseases**	5 point Likert Scale, ranging from Strongly agree to Strongly disagree, option for "I do not wish to answer"
**I try to understand parents' hesitations**	5 point Likert Scale, ranging from Strongly agree to Strongly disagree, option for "I do not wish to answer"
**I inform the parents, but the choice is theirs**	5 point Likert Scale, ranging from Strongly agree to Strongly disagree, option for "I do not wish to answer"

**I am pushing for vaccinations**	5 point Likert Scale, ranging from Strongly agree to Strongly disagree, option for "I do not wish to answer"
**The decision to vaccinate is the parents' responsibility**	5 point Likert Scale, ranging from Strongly agree to Strongly disagree, option for "I do not wish to answer"
**RECOMMENTATIONS AND OTHER QUESTIONS**	
**How often do you recommend vaccines that are NOT included in the mandatory immunization schedule?**	Always<br>Often<br>Rarely Never<br>I cannot answer
**Please chose which are your three main preferred sources of information?**	Official health organizations and institutions<br>Professional associations<br>Vaccine producers (pharmaceutical companies)<br>Specialized medical websites<br>Other general practitioners<br>Medical Journals<br>Social media groups for medics<br>Mesia<br>Other. Please, specify…
**Which of the following would most improve your work? (up to 3 answers, multiple)**	Electronic system for tracking vaccination status<br>Automated messages to patients to remind them of their vaccination dates<br>Special procedure for payment by patients for consultations on vaccines<br>A hotline to give information to GPs about vaccines<br>Electronic resources to provide new information for GPs about vaccines<br>Leaflets on the benefits and risks of each vaccine intended for GPs<br>Leaflets about the benefits and risks of each vaccine intended for patients<br>Public campaigns for the benefit of vaccines<br>Other. Please, specify…

Table S2. Relationships between Factor mean scores and general characteristics of the practice. ANOVA analysis. Mean factor scores and Std. Deviation presented.

Question		Factor mean scores

Type of settlement		Factor1 MEAN (Active communicator)	Factor2 MEAN (Restrictive communicator)	Factor3 MEAN (Informing communicator)	Factor4 MEAN (Strained communicator)
F		2.185	22.926	12.09	8.993
Sig		0.09	0.000	0.000	0.000
The capital (Sofia)	Mean	1.6545	2.6587	1.7374	2.1094
	N	66	63	66	64
	Std. Deviation	0.63976	1.27894	0.95634	0.85202
Regional city	Mean	1.6115	2.2899	1.6643	2.3406
	N	139	138	141	138
	Std. Deviation	0.59504	1.20342	0.90105	1.06192
Small towns	Mean	1.4737	3.2684	1.1803	2.9062
	N	95	95	98	96
	Std. Deviation	0.30604	1.27957	0.43122	1.17274
Village	Mean	1.6383	3.7766	1.1884	2.2391
	N	47	47	46	46
	Std. Deviation	0.36508	1.05711	0.635	1.16304
Total	Mean	1.5856	2.8324	1.4805	2.4419
	N	347	343	351	344
	Std. Deviation	0.51601	1.33291	0.81271	1.1103
**Age**		**Factor1 MEAN (Active communicator)**	**Factor2 MEAN (Restrictive communicator)**	**Factor3 MEAN (Informing communicator)**	**Factor4 MEAN (Strained communicator)**
F		0.087	0.83	1.031	2.048
Sig		0.967	0.478	0.379	0.107
31-40	Mean	1.5091	2.6364	1.3636	1.6818
	N	11	11	11	11
	Std. Deviation	0.56825	1.55066	0.64039	0.71668
41-50	Mean	1.5889	2.7364	1.4906	2.4167
	N	54	55	53	54

Question		Factor mean scores			
**Type of settlement**		**Factor1 MEAN (Active communicator)**	**Factor2 MEAN (Restrictive communicator)**	**Factor3 MEAN (Informing communicator)**	**Factor4 MEAN (Strained communicator)**
	Std. Deviation	0.60115	1.45569	0.82328	1.03135
51-60	Mean	1.5913	2.943	1.5611	2.5219
	N	161	158	161	160
	Std. Deviation	0.51203	1.2794	0.88461	1.12623
over 61	Mean	1.5863	2.7155	1.3962	2.4174
	N	117	116	122	115
	Std. Deviation	0.48261	1.31913	0.72516	1.13155
Total	Mean	1.5866	2.8221	1.4861	2.4426
	N	343	340	347	340
	Std. Deviation	0.51702	1.33036	0.81574	1.1086
**Size of the practice**		**Factor1 MEAN (Active communicator)**	**Factor2 MEAN (Restrictive communicator)**	**Factor3 MEAN (Informing communicator)**	**Factor4 MEAN (Strained communicator)**
F		0.597	0.419	0.375	1.178
sig		0.665	0.795	0.827	0.32
1-1000	Mean	1.6361	2.8729	1.5269	2.275
	N	61	59	62	60
	Std. Deviation	0.60167	1.3696	0.89228	1.01441
1001-2000	Mean	1.5941	2.8413	1.4294	2.4532
	N	170	167	170	171
	Std. Deviation	0.51781	1.33318	0.75928	1.14949
2001-3000	Mean	1.5718	2.8837	1.5249	2.5542
	N	85	86	87	83
	Std. Deviation	0.50252	1.35836	0.86211	1.07355
3001-4000	Mean	1.4667	2.4333	1.6	2.2333

Question		Factor mean scores			
**Type of settlement**		**Factor1 MEAN (Active communicator)**	**Factor2 MEAN (Restrictive communicator)**	**Factor3 MEAN (Informing communicator)**	**Factor4 MEAN (Strained communicator)**
	N	15	15	15	15
	Std. Deviation	0.34365	1.06682	0.77868	1.13179
over 4001	Mean	1.4364	3	1.5455	2.9091
	N	11	11	11	11
	Std. Deviation	0.25009	1.28452	1.01404	1.09129
Total	Mean	1.5854	2.8447	1.4821	2.4515
	N	342	338	345	340
	Std. Deviation	0.51706	1.32997	0.81714	1.10703
**Percentage of children in practice**		**Factor1 MEAN (Active communicator)**	**Factor2 MEAN (Restrictive communicator)**	**Factor3 MEAN (Informing communicator)**	**Factor4 MEAN (Strained communicator)**
F		0.593	2.95	2.096	0.575
Sig		0.553	0.054	0.125	0.563
Up to 20%	Mean	1.5871	2.9438	1.4612	2.4206
	N	170	169	172	170
	Std. Deviation	0.52359	1.33344	0.81915	1.16069
21 - 40%	Mean	1.5361	3	1.3196	2.5556
	N	72	70	73	72
	Std. Deviation	0.40812	1.36997	0.54557	1.15538
Over 41%	Mean	1.6263	2.5395	1.5812	2.3667
	N	76	76	78	75
	Std. Deviation	0.5439	1.26429	0.88996	0.95625
Total	Mean	1.5849	2.8587	1.4582	2.4385
	N	318	315	323	317
	Std. Deviation	0.50439	1.33362	0.7876	1.11314

Table S3. Relationships between factors mean scores and general characteristics of the practice. Mean factor scores and St. Deviation displayed.

Factor		Question				
	Sig	Type of the GPs' practice	N	Mean	Std. Deviation	Std. Error Mean
Factor1 (MEAN) (Active communicator)	0.911	Individual	310	1.5845	0.5045	0.02865
		Group	37	1.5946	0.61189	0.10059
Factor2 (MEAN) (Restrictive communicator)	0.072	Individual	306	2.8775	1.30843	0.0748
		Group	37	2.4595	1.48781	0.2446
Factor3 (MEAN) (Informing communicator)	0.812	Individual	314	1.4841	0.81862	0.0462
		Group	37	1.4505	0.77067	0.1267
Factor4 (MEAN) (Strained communicator)	0.057	Individual	306	2.482	1.1252	0.06432
		Group	38	2.1184	0.93313	0.15137
		**Gender**	**N**	**Mean**	**Std. Deviation**	**Std. Error Mean**
Factor1 (MEAN) (Active communicator)	0.575	Man	101	1.6009	0.54065	0.05380
		Women	246	1.5756	0.50634	0.03228
Factor2 (MEAN) (Restrictive communicator)	0.02	Man	101	3.1832	1.37427	0.13674
		Women	242	2.686	1.29014	0.08293
Factor3 (MEAN)	0.311	Man	102	1.4118	0.8221	0.0814

Factor		Question				
	**Sig**	**Type of the GPs' practice**	**N**	**Mean**	**Std. Deviation**	**Std. Error Mean**
**(Informing communicator)**						
		Women	249	1.5087	0.8088	0.05126
**Factor4 (MEAN) (Strained communicator)**	**0.677**	Man	102	2.4804	1.12997	0.11188
		Women	242	2.4256	1.10386	0.07096
		**Specialty – pediatric**	**N**	**Mean**	**Std. Deviation**	**Std. Error Mean**
**Factor1 (MEAN) (Active communicator)**	**0.923**	Not pediatric	240	1.5875	0.48888	0.03156
		Pediatric	107	1.5813	0.57464	0.05555
**Factor2 (MEAN) (Restrictive communicator)**	**0.035**	Not pediatric	236	2.9343	1.30422	0.0849
		Pediatric	107	2.6075	1.37365	0.1328
**Factor3 (MEAN) (Informing communicator)**	**0.002***	Not pediatric	242	1.3829	0.72772	0.04678
		Pediatric	109	1.6972	0.9434	0.09036
**Factor4 (MEAN) (Strained communicator)**	**0.796**	Not pediatric	240	2.4521	1.13926	0.07354
		Pediatric	104	2.4183	1.04536	0.10251
		**Specialty – internal medicine**	**N**	**Mean**	**Std. Deviation**	**Std. Error Mean**
**Factor1 (MEAN) (Active communicator)**	**0.707**	Not internal medicine	261	1.5916	0.5413	0.03351
		Internal medicine	86	1.5674	0.43262	0.04665

	Factor	Question				
	Sig	Type of the GPs' practice	N	Mean	Std. Deviation	Std. Error Mean
**Factor2 (MEAN) (Restrictive communicator)**	**0.635**	Not internal medicine	257	2.8521	1.33739	0.08342
		Internal medicine	86	2.7733	1.32545	0.14293
**Factor3 (MEAN) (Informing communicator)**	**0.148**	Not internal medicine	264	1.4444	0.79994	0.04923
		Internal medicine	87	1.59	0.84557	0.09065
**Factor4 (MEAN) (Strained communicator)**	**0,238***	Not internal medicine	258	2.4806	1.13813	0.07086
		Internal medicine	86	2.3256	1.01971	0.10996
		**Specialty – general medicine**	**N**	**Mean**	**Std. Deviation**	**Std. Error Mean**
**Factor1 (MEAN) (Active communicator)**	**0.482**	Not general medicine	122	1.6131	0.55966	0.05067
		General medicine	225	1.5707	0.49138	0.03276
**Factor2 (MEAN) (Restrictive communicator)**	**0.004**	Not general medicine	121	2.5496	1.33761	0.1216
		General medicine	222	2.9865	1.30776	0.08777
**Factor3 (MEAN) (Informing communicator)**	**0.00***	Not general medicine	126	1.7037	0.87048	0.07755
		General medicine	225	1.3556	0.75198	0.05013
**Factor4 (MEAN) (Strained communicator)**	**0.244**	Not general medicine	121	2.3471	1.08751	0.09886
		General medicine	223	2.4933	1.12153	0.0751

*Equal variances not assumed

Table S4. Parental hesitancy and attitudes towards vaccines. ANOVA analysis.

**Do the parents of children from your practice have doubts regarding the administration of vaccines from the mandatory children's immunization calendar?**		**Factor1 MEAN (Active communicator)**	**Factor2 MEAN (Restrictive communicator)**	**Factor3 MEAN (Informing communicator)**	**Factor4 MEAN (Strained communicator)**
		7.626	4.41	2.227	12.512
sig		0.001	0.013	0.109	0.000
		Factor1MEAN	Factor2_mean	Factor3MEAN	Factor4MEAN
Yes, often	Mean	1.8085	3.375	1.3958	1.7979
	N	47	44	48	47
	Std. Deviation	0.59557	1.33	0.8155	0.83834
Yes, rarely	Mean	1.6011	2.7201	1.5663	2.4293
	N	184	184	186	184
	Std. Deviation	0.49412	1.25523	0.87375	0.98231
No	Mean	1.4707	2.8043	1.3789	2.7301
	N	116	115	117	113
	Std. Deviation	0.48652	1.41374	0.6933	1.28528
Total	Mean	1.5856	2.8324	1.4805	2.4419
	N	347	343	351	344
	Std. Deviation	0.51601	1.33291	0.81271	1.1103
**In your practice as a General Practitioner in the last 5 years, have there been any cases of illnesses from diseases against which mandatory immunizations from the children's immunization calendar in Bulgaria are currently applied?**		**Factor1 MEAN (Active communicator)**	**Factor2 MEAN (Restrictive communicator)**	**Factor3 MEAN (Informing communicator)**	**Factor4 MEAN (Strained communicator)**
F		1.317	0.722	6.059	1.488
sig		0.269	0.487	0.003	0.227
Yes, without administered vaccine	Mean	1.5	2.875	2.1458	2
	N	16	16	16	16
	Std. Deviation	0.41952	1.53297	1.38761	0.98319
Yes, with administered vaccine	Mean	1.775	3.2188	1.6	2.3125

Do the parents of children from your practice have doubts regarding the administration of vaccines from the mandatory children's immunization calendar?		Factor1 MEAN (Active communicator)	Factor2 MEAN (Restrictive communicator)	Factor3 MEAN (Informing communicator)	Factor4 MEAN (Strained communicator)
	N	16	16	15	16
	Std. Deviation	0.80953	1.21063	0.74748	1.23659
No	Mean	1.5803	2.8103	1.4417	2.4712
	N	315	311	320	312
	Std. Deviation	0.50146	1.32933	0.765	1.10791
Total	Mean	1.5856	2.8324	1.4805	2.4419
	N	347	343	351	344
	Std. Deviation	0.51601	1.33291	0.81271	1.1103
**In your practice as a GP in the last 5 years, have there been any cases of serious adverse effects following compulsory vaccination of children that were potentially associated with hospitalization or disability?**		**Factor1 MEAN (Active communicator)**	**Factor2 MEAN (Restrictive communicator)**	**Factor3 MEAN (Informing communicator)**	**Factor4 MEAN (Strained communicator)**
F		0.014	2.356	1.095	2.084
sig		0.907	0.126	0.296	0.15
Yes	Mean	1.6	3.3056	1.6667	2.0588
	N	18	18	20	17
	Std. Deviation	0.59409	1.53526	0.91127	0.8639
No	Mean	1.5854	2.8117	1.4707	2.4555
	N	328	324	330	326
	Std. Deviation	0.5131	1.31674	0.80734	1.11508
Total	Mean	1.5861	2.8377	1.4819	2.4359
	N	346	342	350	343
	Std. Deviation	0.51666	1.33116	0.81346	1.10632
**Has there been a change in parents' vaccine attitudes about mandatory childhood vaccines since the start of the Covid-19 pandemic?**		**Factor1 MEAN (Active communicator)**	**Factor2 MEAN (Restrictive communicator)**	**Factor3 MEAN (Informing communicator)**	**Factor4 MEAN (Strained communicator)**
F		1.495	1.549	0.362	9.051
sig		0.216	0.202	0.78	0.000

**Do the parents of children from your practice have doubts regarding the administration of vaccines from the mandatory children's immunization calendar?**		**Factor1 MEAN (Active communicator)**	**Factor2 MEAN (Restrictive communicator)**	**Factor3 MEAN (Informing communicator)**	**Factor4 MEAN (Strained communicator)**
Yes, there are less vaccine hesitant parents	Mean	1.555	2.575	1.4167	2.3537
	N	40	40	44	41
	Std. Deviation	0.59137	1.25856	0.71211	1.10817
Yes, there are more vaccine hesitant parents	Mean	1.6716	3.0421	1.5278	2.0189
	N	109	107	108	106
	Std. Deviation	0.4948	1.30514	0.86122	0.88889
No	Mean	1.5417	2.7787	1.4821	2.681
	N	175	174	177	174
	Std. Deviation	0.52569	1.33263	0.83852	1.15064
I can not answer	Mean	1.5652	2.7045	1.3636	2.7391
	N	23	22	22	23
	Std. Deviation	0.35498	1.5404	0.51341	1.17618
Total	Mean	1.5856	2.8324	1.4805	2.4419
	N	347	343	351	344
	Std. Deviation	0.51601	1.33291	0.81271	1.1103

Table S5. Communication practices (difficulties in communication and recommendations of non-mandatory vaccines). ANOVA analysis.

**To what extent do you have difficulty communicating with parents regarding the vaccination of their children?**		**Factor1 MEAN (Active communicator)**	**Factor2 MEAN (Restrictive communicator)**	**Factor3 MEAN (Informing communicator)**	**Factor4 MEAN (Strained communicator)**
F		5.781	2.059	0.48	16.484
sig		0.001	0.105	0.696	0.000
To a great extent	Mean	1.9125	3.5938	1.3922	1.4706
	N	16	16	17	17
	Std. Deviation	0.47311	1.14337	0.66911	0.92653
To some extent	Mean	1.6172	2.9	1.3785	2.1207
	N	58	55	59	58

To what extent do you have difficulty communicating with parents regarding the vaccination of their children?		Factor1 MEAN (Active communicator)	Factor2 MEAN (Restrictive communicator)	Factor3 MEAN (Informing communicator)	Factor4 MEAN (Strained communicator)
	Std. Deviation	0.43413	1.24499	0.64759	0.82881
To a small extent	Mean	1.6785	2.8224	1.5121	2.1881
	N	107	107	110	109
	Std. Deviation	0.5304	1.30176	0.8334	0.89698
I do not experience any difficulties in communicating with parents	Mean	1.4831	2.7424	1.5051	2.8344
	N	166	165	165	160
	Std. Deviation	0.51506	1.38327	0.86611	1.20105
Total	Mean	1.5856	2.8324	1.4805	2.4419
	N	347	343	351	344
	Std. Deviation	0.51601	1.33291	0.81271	1.1103
**How often do you recommend vaccines that are NOT included in the mandatory immunization schedule?**		**Factor1 MEAN (Active communicator)**	**Factor2 MEAN (Restrictive communicator)**	**Factor3 MEAN (Informing communicator)**	**Factor4 MEAN (Strained communicator)**
F		6.513	2.63	0.946	2.911
sig		0.000	0.034	0.437	0.022
Always	Mean	1.4588	2.6893	1.3556	2.6667
	N	102	103	105	102
	Std. Deviation	0.35554	1.38641	0.76414	1.09303
Often	Mean	1.5567	2.9444	1.5438	2.4387
	N	157	153	160	155
	Std. Deviation	0.49475	1.26251	0.8348	1.09505
Rarely	Mean	1.7231	2.5781	1.5104	2.1797
	N	65	64	64	64
	Std.	0.57738	1.33992	0.73695	1.11756

To what extent do you have difficulty communicating with parents regarding the vaccination of their children?		Factor1 MEAN (Active communicator)	Factor2 MEAN (Restrictive communicator)	Factor3 MEAN (Informing communicator)	Factor4 MEAN (Strained communicator)
	Deviation				
Never	Mean	2.0222	3.5833	1.5686	2.3889
	N	18	18	17	18
	Std. Deviation	0.87283	1.29762	1.18301	1.18266
I cannot answer	Mean	1.72	2.9	1.4	1.5
	N	5	5	5	5
	Std. Deviation	0.30332	1.51658	0.36515	0.5
Total	Mean	1.5856	2.8324	1.4805	2.4419
	N	347	343	351	344
	Std. Deviation	0.51601	1.33291	0.81271	1.1103
